# Deletion of aquaporin-4 in APP/PS1 mice exacerbates brain Aβ accumulation and memory deficits

**DOI:** 10.1186/s13024-015-0056-1

**Published:** 2015-11-02

**Authors:** Zhiqiang Xu, Na Xiao, Yali Chen, Huang Huang, Charles Marshall, Junying Gao, Zhiyou Cai, Ting Wu, Gang Hu, Ming Xiao

**Affiliations:** Jiangsu Key Laboratory of Neurodegeneration, Nanjing Medical University, 140 Hanzhong Road, Nanjing, Jiangsu 210029 China; Department of Rehabilitation Sciences, University of Kentucky Center of Excellence in Rural Health, Hazard, KY USA; Department of Neurology, Renmin Hospital, Hubei University of Medicine, Shiyan, Hubei China; Department of Neurology, The First Affiliated Hospital of Nanjing Medical University, Nanjing, Jiangsu China

**Keywords:** Alzheimer’s disease, Amyloid-beta, Astrocytes, AQP4, Paravascular pathway

## Abstract

**Background:**

Preventing or reducing amyloid-beta (Aβ) accumulation in the brain is an important therapeutic strategy for Alzheimer’s disease (AD). Recent studies showed that the water channel aquaporin-4 (AQP4) mediates soluble Aβ clearance from the brain parenchyma along the paravascular pathway. However the direct evidence for roles of AQP4 in the pathophysiology of AD remains absent.

**Results:**

Here, we reported that the deletion of AQP4 exacerbated cognitive deficits of 12-moth old APP/PS1 mice, with increases in Aβ accumulation, cerebral amyloid angiopathy and loss of synaptic protein and brain-derived neurotrophic factor in the hippocampus and cortex. Furthermore, AQP4 deficiency increased atrophy of astrocytes with significant decreases in interleukin-1 beta and nonsignficant decreases in interleukin-6 and tumor necrosis factor-alpha in hippocampal and cerebral samples.

**Conclusions:**

These results suggest that AQP4 attenuates Aβ pathogenesis despite its potentially inflammatory side-effects, thus serving as a promising target for treating AD.

**Electronic supplementary material:**

The online version of this article (doi:10.1186/s13024-015-0056-1) contains supplementary material, which is available to authorized users.

## Background

Alzheimer’s disease (AD) is a common neurodegenerative disorder, characterized by progressive cognitive dysfunction. To this point, there are no effective therapeutic strategies for this devastating disease [1]. The pathogenesis of AD remains unclear, but various mechanisms cause an imbalance between amyloid-beta (Aβ) production and clearance. This results in increased levels of soluble Aβ and final plaque accumulation within the brain parenchyma, which is a key step in the onset and development of AD [2]. Identifying potential pathways that are responsible for clearance of soluble Aβ from the brain is an important strategy for the treatment of AD.

Evidence from a series of recent experiments reveals that the paravascular pathway facilitates the clearance of interstitial solutes (ISF), including a large proportion of soluble Aβ, from the brain parenchyma [3–6]. Impairment in ISF drainage along perivascular spaces with amyloid deposits occurs in transgenic mouse models of AD, suggesting that improving Aβ clearance along the paravascular pathway may provide a feasible therapeutic approach to control the progression of AD [7].

The water channel aquaporin-4 (AQP4) is primarily localized to perivascular astrocyte endfeet and astrocyte membranes facing the pia mater, helping to maintain water homeostasis in the central nervous system [8]. Altered AQP4 expression and localization in reactive astrocytes has been observed in patients with AD and several transgenic mouse models of AD [9–12]. Furthermore, animal experiments demonstrate that AQP4 is responsible for the bulk ISF flow that drives the clearance of interstitial Aβ from the brain parenchyma [5, 6]. These results suggest that the application of AQP4 agonists or opening agents can promote brain interstitial Aβ clearance, and thus delay or even counter the progression of AD.

Reactive astrogliosis surrounding amyloid plaques is one of the neuropathological hallmarks of AD. Attenuating astrocyte activation has been shown to accelerate plaque pathogenesis in mouse AD model [13]. Previous studies suggested that AQP4 deficiency alters astrocyte reactivation after traumatic brain or spinal cord injuries and chemical agents-induced neurodegeneration [14–17]. We also found that genetic deletion of AQP4 reduces Aβ-induced activation of cultured astrocytes, which is associated with a reduction in the uptake of Aβ [18]. Moreover, AQP4 is implicated in proinflammatory features of astrocytes, which is an aggravating factor in the AD pathology [19, 20]. Therefore, clarifying the overall role of AQP4 in the AD pathophysiological process should be a premise for therapeutic strategies of AD.

In this study, we successfully established AQP4 gene knockout (AQP4^−/−^) Aβ precursor protein/presenilin 1 (APP/PS1) transgenic mice and demonstrated that the deletion of AQP4 has a tendency to reduce neuroinflammation, but aggravates brain Aβ accumulation, subsequently exacerbating synaptic protein loss, astrocyte atrophy and cognitive dysfunction in 12-month old APP/PS1 mice.

## Results

### AQP4 deficiency aggravated cognitive impairment in APP/PS1 mice

**Fig. 1:**
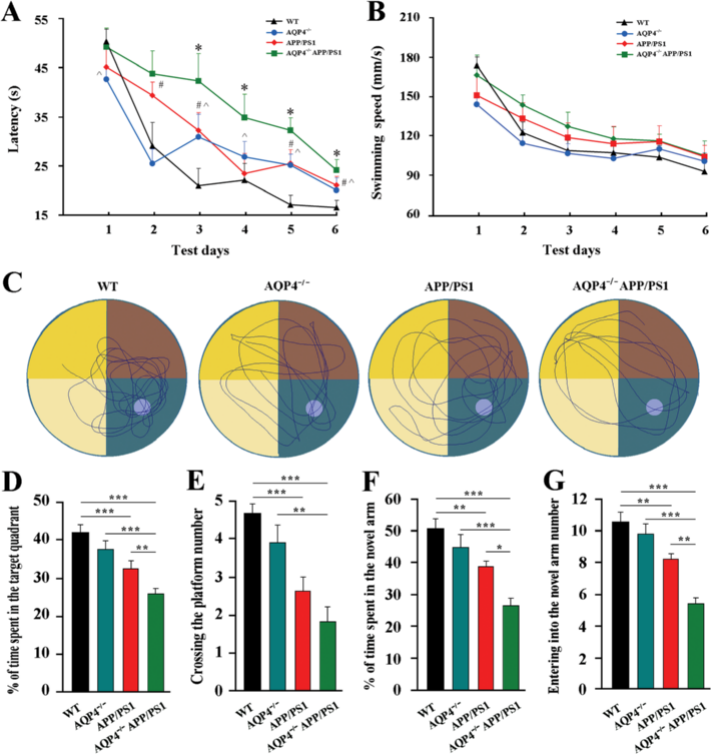
AQP4 deficiency aggravated cognitive impairment in 12 month-old APP/PS1 mice. **a** The mean escape latency, and **b** swimming speed during the hidden platform training period of the Morris water maze test. **c** Tracings of the typical swim patterns. **d** The percentage of time spent in the target quadrant. **e** The number of crossing the platform area. **f** The percentage of time spent in the novel arm. **g** The number of entries into the novel arm in the Y-maze. Data represent mean ± SEM from 9 to 11 mice (6–8 female, and 3–5 male) per group. Data in Fig. 1a and b were analyzed by repeated-measures ANOVA with post hoc Student-Newman-Keuls test, *P < 0.05, AQP4^−/−^APP/PS1 mice *vs* APP/PS1 mice; #*P* < 0.05, APP/PS1 mice *vs* WT mice; ^*P* < 0.05, AQP4^−/−^ mice *vs* WT mice. Data in Fig. 1d-g were analyzed by ANOVA with post hoc Student-Newman-Keuls test. *P < 0.05; **P < 0.01; ***P < 0.001

We examined the spatial learning and memory of wild-type (WT), AQP4^−/−^, APP/PS1 and AQP4^−/−^APP/PS1 mice at 12 months of age using the Morris water maze test. These genotype mice showed different performances during the hidden platform tests (Fig. 1a). For WT mice, the escape latency progressively decreased over the 6 days of training. AQP4^−/−^ mice had an easier time finding the hidden platform during the early stage of the training (1 day), but seemed to forgot quickly during the latter stages (3–6 days), indicating impaired memory consolidation [21]. APP/PS1 mice exhibited impaired learning ability, compared to WT mice. Genetic deletion of AQP4 further exacerbated cognitive impairment in APP/PS1 mice, reflected by extended escape latency during the entire training periods. Swimming speed was equivalent among the four groups, suggesting that their differing time spent locating the underwater platform was unrelated to motor ability (Fig. 1b). In the probe test, swimming paths of WT mice were highly concentrated in the target quadrant. APP/PS1 mice and AQP4^−/−^ mice tended to search in a larger area of the pool, but still preferred the target quadrant. In contrast, AQP4^−/−^APP/PS1 mice frequently swam randomly in each quadrant without any purpose (Fig. 1c). The percentage of time spent in the target quadrant, and numbers of platform area crossings in AQP4^−/−^APP/PS1 mice were lowest among the four groups (Fig. 1d, e). Genetic deletion of AQP4 exaggerating spatial memory deficits in APP/PS1 mice was further confirmed by the Y-maze test, with the lowest time exploring the novel arm and the lowest numbers of the novel arm entries during the retention trial (Fig. 1f, g).

### AQP4 deficiency increased brain Aβ accumulation and CAA in APP/PS1 mice without altering the expression levels of proteins associated with Aβ formation and degradation

**Fig. 2:**
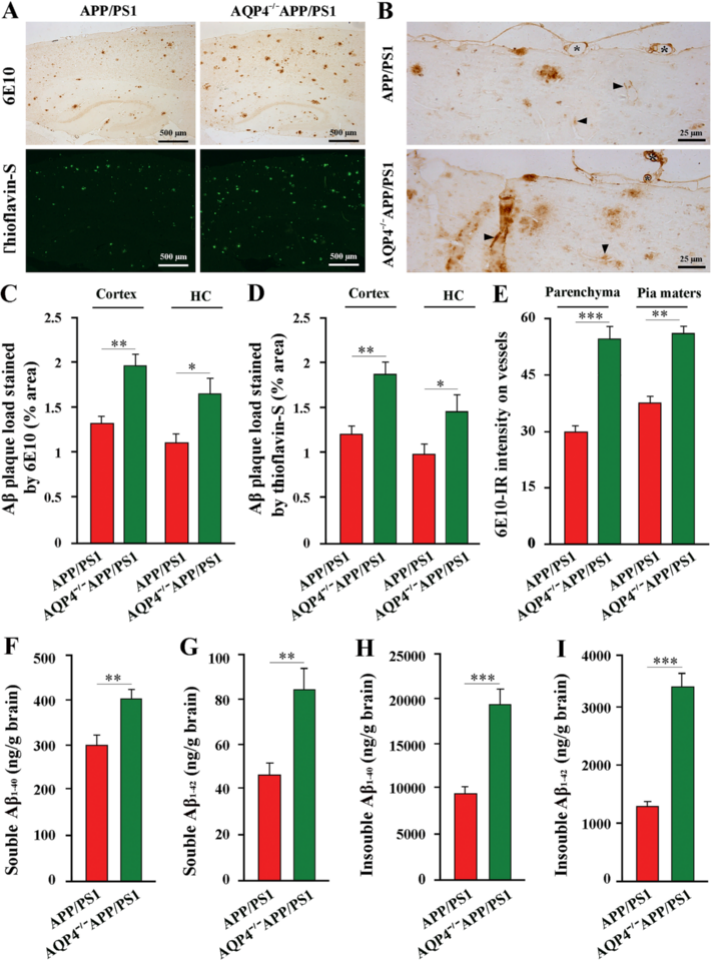
AQP4 deficiency increased brain Aβ accumulation and CAA in 12 month-old APP/PS1 mice. **a** Aβ deposition in the hippocampus and cortex stained by 6E10 and Thioflavin-S. **b** High magnification micrographs of 6E10 immunostaining. There was increased amyloid deposition along small and large vessels in the brain parenchyma (arrowheads) and leptomeningeal vessels (stars) of AQP4^−/−^APP/PS1 mice, compared to APP/PS1 controls. **c** The area percentage of 6E10-positive Aβ plaque load in the hippocampus (HC) and cerebral cortex. **d** The area percentage of Thioflavin-S-positive Aβ plaque load in the hippocampus (HC) and cerebral cortex. **e** 6E10-immunoreactive (IR) intensity on the cortical vessels and leptomeningeal vessels. **f-i** ELISA analysis of soluble and insoluble Aβ_1–40_ and Aβ_1–42_ in the brain samples. Data represent mean ± SEM from 5 to 6 mice (3–4 female, and 1–2 male) per group. The statistical analysis was performed by Student’s *t*-test. *P < 0.05; **P < 0.01; ***P < 0.001

The previous study revealed that AQP4 deficiency impairs the clearance of intraparenchymal injection of exogenous Aβ peptide [5]. In the present study, we addressed the long-term contribution of AQP4 to the clearance of endogenous Aβ in APP/PS1 mouse brain. In 12-month old AQP4^−/−^APP/PS1 mice, Thioflavin-S positive fibrillar plaques and 6E10-immunopositive diffuse plaques occupied a great deal of the hippocampal and cortical areas, compared to APP/PS1 mice (Fig. 2a, c, d). In addition to the brain parenchyma, Aβ deposition manifested in cortical and leptomeningeal vessels as cerebral amyloid angiopathy (CAA), which was also increased in the AQP4^−/−^APP/PS1 mice (Fig. 2b, e). Consistent with increased amyloid load, ELISA analysis demonstrated that AQP4 deficiency increased both soluble and insoluble Aβ_1–40_ and Aβ_1–42_ levels in APP/PS1 mouse brain (Fig. 2f-i).

**Fig. 3:**
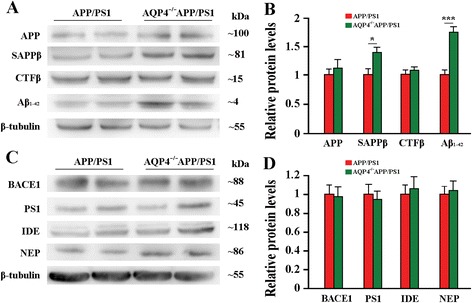
AQP4 deficiency did not alter expression of brain Aβ formation and degradation-related proteins in 12 month-old APP/PS1 mice. **a** Representative bands of Western bolt and **b** densitometry analysis of protein levels of APP and its proteolytic amyloidogenic fragments. **c** Representative bands of Western bolt and **d** densitometry analysis of protein levels of the biochemical components involved in the amyloid production (BACE1 and PS1) and degradation (IDE and NEP). Data represent mean ± SEM from 5 to 6 mice (3–4 female, and 1–2 male) per group. The statistical analysis was performed by Student’s *t*-test

To further investigate the molecular mechanisms of the effects of AQP4 deficiency on plaque pathogenesis, we examined the expression of proteins involved in Aβ synthesis and degradation in the cerebral cortex and hippocampus lysates. APP is processed first by the β-secretase (β-site amyloid precursor protein-cleaving enzyme 1, BACE1) to form the soluble peptide APP β (SAPPβ), and transmembrane peptide C-terminal fragments (CTFβ) [22]. The latter are cleaved by the γ-secretase complex, including presenilin 1 (PS1), to form Aβ peptides, which are subsequently released into the extracellular space [22]. AQP4^−/−^APP/PS1 mice demonstrated significant increases in levels of Aβ_1–42_ and SAPPβ, but no significant changes in APP, CTFβ, BACE1 and PS1 (Fig. 3a-d), suggesting that AQP4 gene deletion did not affect APP production and processing, but selectively impaired clearance of its soluble proteolytic fragments from the brain. In addition, insulin degrading enzyme (IDE) and neprilysin (NEP), the two major Aβ-degrading enzymes in the brain [23], showed no significant changes in AQP4^−/−^APP/PS1 mice (Fig. 3c, d) (Additional file 1: Figure S1). Taken together, these data indicate that AQP4 deficiency could be impair rapid transport of water-mediated Aβ clearance from the brain parenchyma, subsequently increasing the Aβ burden in APP/PS1 mouse brain without altering the expression levels of Aβ formation and degradation-related proteins.

### AQP4 deficiency accelerated the progression of astrocyte pathology in APP/PS1 mouse brain

**Fig. 4:**
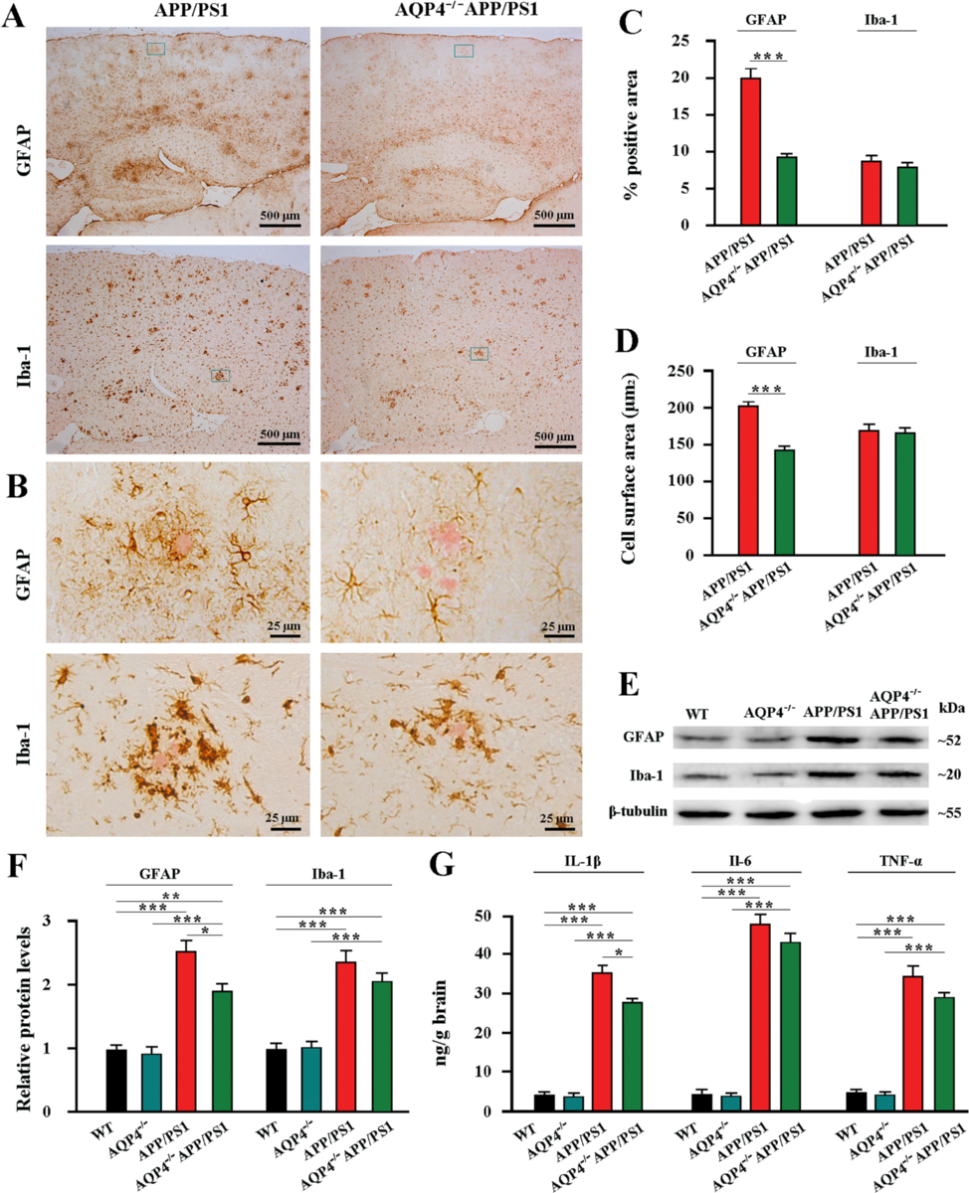
AQP4 deficiency increased astrocyte atrophy with attenuation of reactive gliosis and neuroinflammation in 12 month-old APP/PS1 mice. **a** GFAP positive reactive astrogliosis was significantly attenuated in the hippocampus and cerebral cortex of AQP4^−/−^APP/PS1 mice compared with APP/PS1 controls. A mild reduction of iba-1 positive reactive microgliosis also occurred in AQP4^−/−^APP/PS1 mouse brain. **b** The high magnification images showing that Congo Red positive plaques were surrounded by activated GFAP-positive astrocytes, displaying morphological features of hypertrophy, in APP/PS1 mice. In contrast, astrocytes adjacent to plaques exhibited atrophy with weak GFAP expression in AQP4^−/−^APP/PS1 mice. The reactive microgliosis around plaques was mildly reduced in AQP4^−/−^APP/PS1 mice, compared that in APP/PS1 controls. **c** The percentage of GFAP or Iba-1 positive area in the hippocampus and cortex. **d** GFAP-positive surface area per astrocyte and Iba-1-positive surface area per microglial cell surrounding Aβ plaques within the cortex. **e** Representative bands of Western bolt and **f** densitometry analysis of GFAP and Iba-1 protein levels in the hippocampus and cortex. **g** ELISA analysis of IL-1β, IL-6 and TNF-α levels in the hippocampus and cortex. Data represent mean ± SEM from 5 to 6 mice (3–4 female, and 1–2 male) per group. Data in Fig. 4c and d were analyzed by Student’s *t*-test and in Fig. 4f and g by ANOVA with post hoc Student-Newman-Keuls test. *P < 0.05; **P < 0.01; ***P < 0.001

Both activated astrocytes and microglia have been shown to limit deposits of Αβ through phagocytosis and degradation [24, 25]. We addressed whether AQP4 deficiency altered Αβ-related reactive gliosis in the hippocampus and cerebral cortex of APP/PS1 mice at 12 months of age, using glial fibrillary acidic protein (GFAP) and ionized calcium-binding adaptor molecule 1 (Iba-1) immunohistochemistry. Each Αβ plaque was surrounded by GFAP-IR astrocytes and Iba-1-IR microglia in APP/PS1 mice. In contrast, a considerable portion of Αβ plaques lacked GFAP-IR astrocytes surrounding in AQP4^−/−^APP/PS1 mice (Fig. 4a, b). A decreased number of Iba-1-IR microglia around Αβ plaques was also observed, especially in the cerebral cortex (Fig. 4a). Quantitative analysis revealed that AQP4^−/−^APP/PS1 mice had a decreased area percentage of GFAP expression compared with APP/PS1 mice, but the difference was not significant for those of Iba-1 (Fig. 4c). Western blot analysis also revealed that AQP4 deficiency significantly attenuated up-regulation of GFAP, but not Iba-1, in the hippocampus and cortex of APP/PS1 mice, as compared to WT and AQP4^−/−^ mice (Fig. 4e, f).

It has also been shown that, apart from reactive hypertrophy, a subpopulation of astrocytes undergo apoptosis or atrophy in AD patients [26, 27] and transgenic AD animal models [28, 29], due to chronic exposure to high concentrations of Aβ. Therefore, we determined whether the reduced reactive astrogliosis was a late stage phenomenon of atrophy of astrocytes. The quantitative analysis revealed that 12-month old AQP4^−/−^APP/PS1 mice had a smaller cellular surface area of GFAP-positive astrocytes surrounding plaques in the cerebral cortex, when compared to age matching APP/PS1 controls (Fig. 4b, d).

Furthermore, different from reduced reactive gliosis at 12 months of age, 6.5 month-old AQP4^−/−^APP/PS1 mice exhibited increases in reactive gliosis, as well as Αβ load, in the hippocampus and cortex, when compared with APP/PS1 controls (Additional file 2: Figure S2). Together, these data suggest that both reactive gliosis and astrocyte atrophy peaked earlier in AQP4^−/−^APP/PS1 mice due to acceleration of AD pathology.

Activated astrocytes and microglia facilitate Αβ clearance, but also mediate inflammation via cytokine production. Thus, reactive gliosis may play a double-edged role in the AD pathology [30, 31]. Previous studies have shown that AQP4 is involved in the release of cytokines by activated astrocytes [19, 20]. Therefore, we examined whether the attenuation of reactive astrogliosis affected the production of inflammatory factors in the hippocampus and cortex of 12-month AQP4^−/−^APP/PS1 mice. APP/PS1 mice had significantly higher levels of interleukin-1 beta (IL-1β), and higher levels, although nonsignficant, of interleukin-6 (IL-6) and tumor necrosis factor-alpha (TNF-α) than AQP4^−/−^APP/PS1 mice (Fig. 4g). There was no difference in baseline levels of IL-1β, IL-6 and TNF-α between WT and AQP4^−/−^ controls.

### Atrophic astrocytes surrounding Aβ plaques decreased LRP1 expression in AQP4^−/−^APP/PS1 mice

**Fig. 5:**
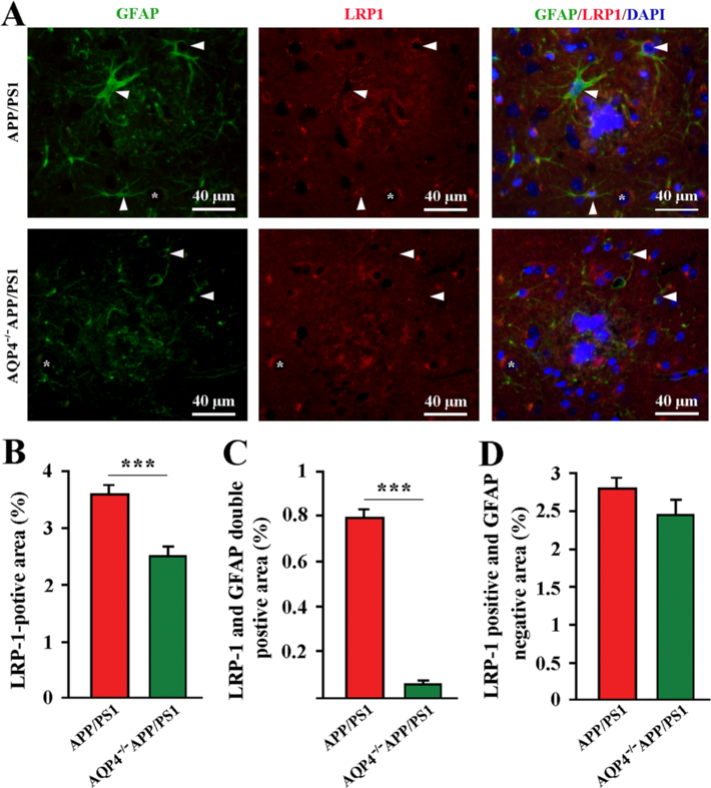
AQP4 deficiency decreased LRP1 expression by astrocytes surrounding Aβ plaques in APP/PS1 mice. **a** Activated GFAP-positive (green) astrocytes surrounding plaque-like structures (blue) clearly expressed LRP1 (red) (arrowheads) in 12-month old APP/PS1 mice. In contrast, in age-matching AQP4^−/−^APP/PS1 mice, GFAP-positive astrocytes around plaques were atrophy with very low immunofluorescent staining of LRP1 (arrowheads). Some cells negative for GFAP (stars), expressed LRP1 in the both gene-type brains. **b** The percentage of LRP1 positive area within a radius of 100 μm to the plaque border. **c** The percentage of LRP1 and GFAP double positive area. **d** The percentage of LRP1 positive, but GFAP negative area. Data represent mean ± SEM from 5 to 6 mice (3–4 female, and 1–2 male) per group. The statistical analysis was performed by Student’s *t*-test. ***P < 0.001

Low density lipoprotein receptor-related protein-1 (LRP1) is expressed by multiple brain cell types, with an important role in ISF soluble Αβ uptake and clearance across the blood–brain-barrier [32, 33]. Our previous studies have shown that AQP4 deficiency reduces uptake of Αβ by cultured astrocytes, which is associated with decreased up-regulation of LRP1 [18]. Consistently, we demonstrated that GFAP-positive astrocytes located around the amyloid plaques underwent atrophy with very low and even undetectable levels of LRP1 expression in 12-month old AQP4^−/−^APP/PS1 mice (Fig. 5a). In contrast, in age-matching APP/PS1 mice, GFAP-positive astrocytes adjacent to Aβ plaques exhibited hypertrophic feature with obvious LRP1 expression (Fig. 5a). The subsquent quantitative analysis revealed that AQP4^−/−^APP/PS1 mice had decreases in the area percentage of LRP1 labeling or LRP1 and GFAP double labeling within a radius of 100 μm to the plaque border, compared with APP/PS1 mice (Fig. 5b, c). However, the area percentage of LRP1 positive and GFAP negative was not different each other (Fig. 5d), further suggesting that astrocyte atrophy is a key factor for reduced LRP1 expression surrounding Aβ plaques in AQP4^−/−^APP/PS1 mice. In addition, hippocampal and cortical neurons highly expressed LRP1, which was not affected by AQP4 deficiency (Additional file 3: Figure S3A, S3C). Western blot also showed that brain LRP1 protein levels were similar between AQP4^−/−^APP/PS1 mice and APP/PS1mice (Additional file 3: Figure S3B, S3D).

### AQP4 deficiency exacerbated decreases in synaptic protein expression and BDNF production in APP/PS1 mouse brain

**Fig. 6:**
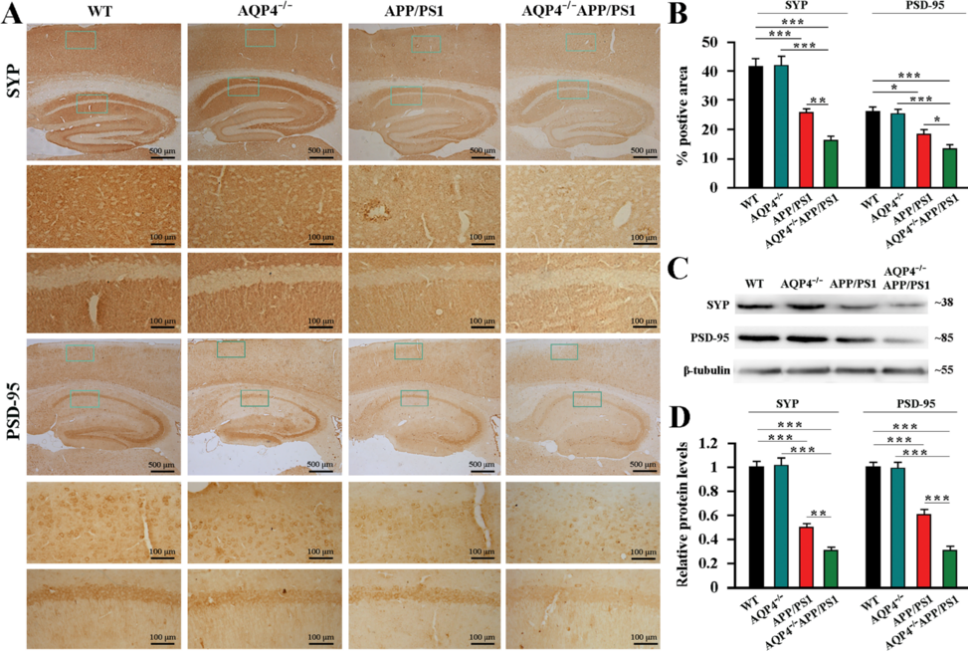
AQP4 deficiency exacerbated decreases in SYP and PSD-95 in 12-month-old APP/PS1 mice. **a** SYP and PSD-95 expression was markedly decreased in the hippocampus and cerebral cortex of AQP4^−/−^APP/PS1 mice, compared to APP/PS1 mice. **b** The percentage of SYP/PSD-95 positive area in the hippocampus and cortex. **c** Representative bands of Western bolt and **d** densitometry analysis of SYP and PSD-95 protein levels in the hippocampus and cortex. Data represent mean ± SEM from 5 to 6 mice (3–4 female, and 1–2 male) per group. The statistical analysis was performed by ANOVA with post hoc Student-Newman-Keuls test. *P < 0.05; **P < 0.01; ***P < 0.001

Synaptic impairment in the hippocampus and cerebral cortex is a main pathological basis for cognitive impairment in AD [2]. Both synaptophysin (SYP) and post-synaptic density protein-95 (PSD-95) are particularly vulnerable to the toxic effects of Αβ, and their loss correlates with cognitive decline in AD [34–36]. Therefore, we determined whether genetic deletion of AQP4 in APP/PS1 mice advanced cognitive impairment was associated with more severe impairment of SYP and PSD-95. Deletion of AQP4 in 12-month old APP/PS1 mice significantly exacerbated loss of SYP and PSD-95 in the hippocampus and cerebral cortex as revealed by both immunohistochemistry (Fig. 6a, b) and Western blot analyses (Fig. 6c, d).

**Fig. 7:**
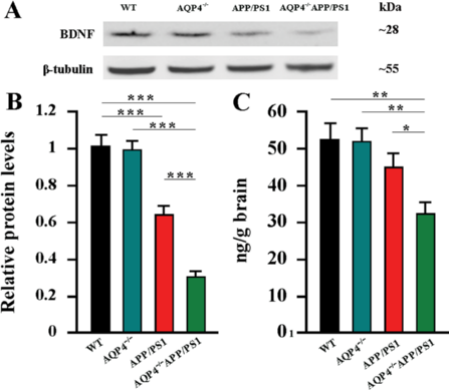
AQP4 deficiency promoted decreases in BDNF production in 12-month old APP/PS1 mice. **a** Representative bands of Western bolt and **b** densitometry analysis of BDNF levels in the hippocampus and cortex. **c** ELISA analysis of BDNF levels in the hippocampus and cortex. Data represent mean ± SEM from 5 to 6 mice (3–4 female, and 1–2 male) per group. The statistical analysis was performed by ANOVA with post hoc Student-Newman-Keuls test. *P < 0.05; **P < 0.01; ***P < 0.001

In addition, brain-derived neurotrophic factor (BDNF) is a key regulator of neuronal survival and synaptic plasticity that are required for the long-term learning and memory [37]. Aβ accumulation has been implicated in decreased BDNF levels by inhibiting activation of CREB, a transcription factor that regulates BDNF expression [38]. Decreased production of BDNF, in turn, exacerbates Aβ-related neurodegeneration in late-onset AD [39]. Consistently, 12-month old AQP4^−/−^APP/PS1 mice had lower levels of BDNF in the hippocampus and cerebral cortex, when compared to APP/PS1 controls, as revealed by Western blot (Fig. 7a, b) and ELISA (Fig. 7c).

### Impaired AQP4 polarization during the pathological progression of APP/PS1 mice

**Fig. 8:**
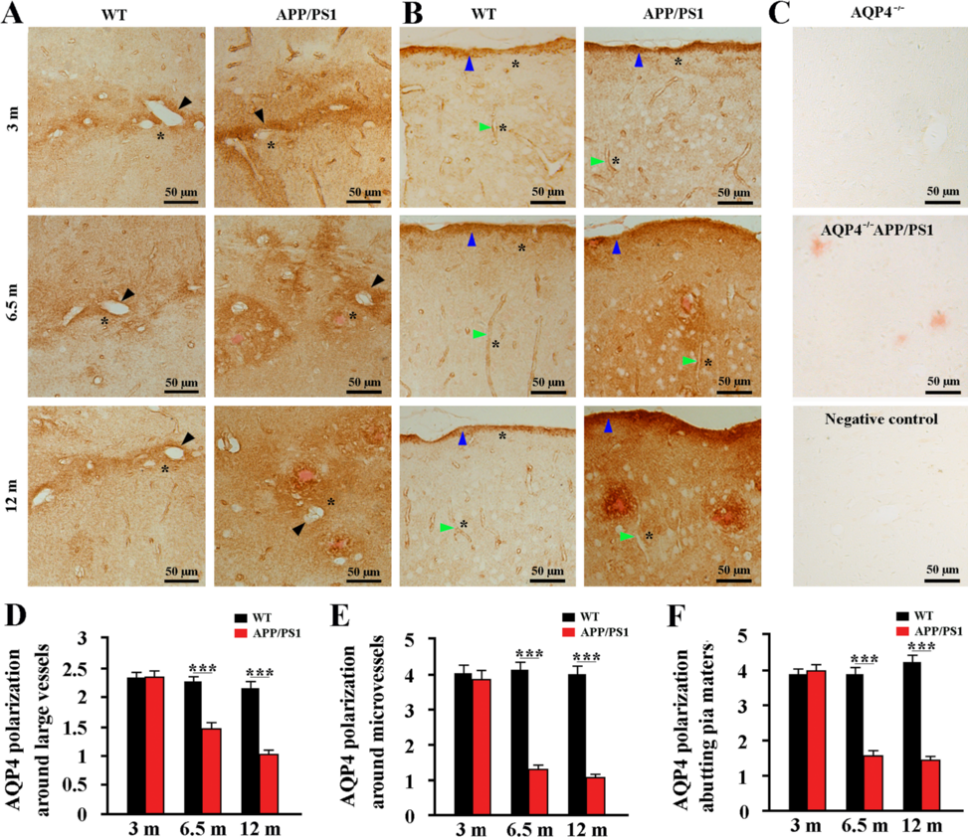
AQP4 polarization was lost during the pathological progression of APP/PS1 mice. Representative AQP4 immunoreactive images of the hippocampus **a** andcerebral cortex **b** of WT mice and APP/PS1 mice at age of 3, 6.5 and 12 months. From 3 to 12 months of age, WT mice showed high AQP4 expression closely abutting the large vessels (black arrows), microvessels (green arrows) and pia surface (blue arrows), but low AQP4 expression in the adjacent parenchyma (indicated by stars). In 3-month-old APP/PS1 mice that had no Aβ plaque disposition, AQP4 expression was slightly increased at the regions immediately abutting large vessels, microvessels and pia maters as well as correspondingly adjacent parenchymal domains. At 6.5 and 12 months of age, AQP4 was markedly increased at parenchymal domains surrounding Aβ plaques, but not significantly changed at the perivascular domains. **c** There was no immunoreactive signal for AQP4 in the hippocampus of AQP4^−/−^ mice and AQP4^−/−^APP/PS1 mice. Brain sections from WT mice were also immuno-negative when the AQP4 antibody were omitted and replaced with an equivalent concentration of normal rabbit serum. Congo Red positive plaques were only observed in the AQP4^−/−^APP/PS1 hippocampus. Quantitative analyses of the AQP4 polarization abutting large vessels **d** (*n* = 16–20 per mouse), microvessels **e** (*n* = 16–20 per mouse) and pia maters **f** (*n* = 4 per mouse). Data represent mean ± SEM from 5 to 6 mice (3–4 female, and 1–2 male) per group. The statistical analysis was performed by Student’s *t*-test. ***P < 0.001

In the health brain, AQP4 is highly expressed in astrocyte processes abutting cerebral microvessels or pia, while its density is comparatively low at astrocyte membrane facing neuropil regions [40]. However, under various pathological conditions, reactive astrogliosis causes mislocalization expression of AQP4, subsequently impairs its polarity at the perivascular endfoot processes and glial limitans beneath the pia mater [3, 10, 12, 41]. Therefore, we investigated whether this pathological change would also happen during the pathological progress of APP/PS1 mouse brain. The immunohistochemical results showed that AQP4 expression was highly polarized in the brain of WT mice at 3, 6.5 and 12 months of age, characterized by high intensity of AQP4-IR products surrounding large vessels and microvessels or facing pia maters, and very low intensity in the adjacent parenchyma. AQP4 expression was slightly increased at the regions immediately abutting large vessels, microvessels and pia maters as well as correspondingly adjacent parenchymal domains in 3-month-old APP/PS1 mice that had no Aβ plaque disposition in the brain. AQP4 expression was significantly increased at parenchymal domains, particularly around Aβ plaques, but unchanged at perivascular domains of 6.5 and 12 month-old APP/PS1 mice. In addition, AQP4 immunoreactivity was significantly increased at the cerebral pia surface, as well as the subpial region (Fig. 8a, b). No AQP4 immunoreactive signal was observed in the brain of AQP4^−/−^ mice and AQP4^−/−^APP/PS1 mice (Fig. 8c). The selective localization of AQP4 to vessels or pia maters was disrupted in APP/PS1 mice at 6.5 and 12 months of age, as confirmed by the quantitative data of AQP4 polarization (Fig. 8d-f).

## Discussion

**Fig. 9:**
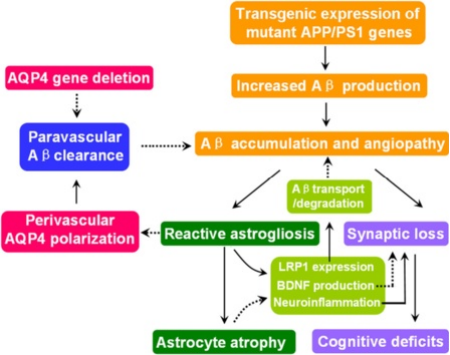
Schematic diagram of AQP4 mitigating plaque pathogenesis in APP/PS1 mice. We propose that genetic deletion of AQP4 that mediates paravascular interstitial Aβ clearance in APP/PS1 mice results in accelerated accumulation of Aβ in brain parenchyma and cerebral blood vessels, and subsequently improves synaptic degeneration and astrocyte atrophy. In addition, extensive reactive astrocytes would impair AQP4 polarity, thus impairing the efficiency Aβ clearance from paravascular space into blood and CSF

Aβ accumulation and aggregation in the extracellular space of the brain is a pathological hall mark of AD. Aβ peptides in the ISF aggregate into higher-order species, such as soluble oligomers and insoluble amyloid plaques, in a concentration-dependent manner. Thus, clearance of accumulated Aβ from the brain parenchyma is a potentially promising therapeutic strategy to delay or even counter Aβ pathology and AD progression [42, 43]. AQP4 is localized to astrocyte processes neighboring cerebral microvessels or pia in a highly polarized manner. This expression feature has been shown to facilitate the efficient clearance of ISF solutes, such as Aβ and Tau, from the brain parenchyma [3, 5]. In the present study, utilizing AQP4^−/−^APP/PS1 mice, we have provided direct evidence for the involvement of AQP4 in Aβ-related pathophysiology. Long-term deficiency of AQP4 increases brain Aβ plaque deposits, CAA, synaptic protein damage and cognitive dysfunction in APP/PS1 mice. Exacerbated Aβ accumulation in AQP4^−/−^APP/PS1 brain also increases astrocyte atrophy with decreases in LRP1 expression, which in turn would reduce the efficiency of Aβ clearance from the brain. Atrophic astrocytes may attenuate their proinflammatory roles, while also reduce BDNF production, thus potentially playing a double-edge role on synaptic degeneration (Fig. 9). These results suggest that the overall contribution of AQP4 in the AD-like pathophysiology of APP/PS1 mice serves a protective function, highlighting a potential role of AQP4 as a therapeutic target for AD.

Accumulating evidence supports the notion that the glial water channel AQP4 is not only responsible for maintaining brain water homeostasis, but also involved in various biological functions of astrocytes such as regulation of extracellular space volume, potassium buffering, calcium signal transduction, neuronal excitability and synaptic plasticity under baseline conditions [40, 44]. For example, deletion of AQP4 in mice impairs BDNF-dependent synaptic plasticity without alterations of synaptic protein expression in the intact brain [45]. Behavioral analyses reveal that AQP4^−/−^ mice have impairment in memory consolidation, rather than short-term memory [21, 45, 46]. The critical role of AQP4 in astrocyte functions is evenly evident in pathological conditions, including cerebral edema [47], intracerebral hemorrhage [48], neuromyelitis optica [49], neuroinflammation [19], and epilepsy [50]. In agreement with this view, the present study revealed that deletion of AQP4 in APP/PS1 mice, but no in WT mice, decreases SYP and PSD-95 expression and BDNF production. Consistently, AQP4 deficiency in the intact mice only affects spatial learning performance during the latter stages of the Morris maze training. In contrast, AQP4 deficiency in APP/PS1 mice causes longer escape latency during the entire training periods. Specifically, deleterious effect of AQP4 deletion on the spatial memory performance in the Morris maze as well as the Y-maze is observed in APP/PS1 mice, but not in WT mice (Fig. 1d-g). These results suggest that AQP4 deficiency increases the vulnerability of memory-related structural and functional impairment under AD-like pathology.

Aβ accumulation is a key pathological source for synaptic loss and cognitive impairment in AD patients and transgenic mice [42, 43]. At very low levels, Aβ peptides act as a normal signal molecule, preventing excessive activation of neurons. When the balance between brain Aβ production and clearance is broken, aggregated Aβ in the extracellular space gradually manifests its neurotoxic effects, finally causing neurodegeneration and cognitive impairment [51]. AQP4 deficiency exacerbates Aβ accumulation in the cortex and hippocampus of APP/PS1 mice, which is the main pathological factor for severe spatial memory deficits, although loss of AQP4 physiological functions itself is also involved in alterations in cognitive performance.

The generation of Aβ from APP is controlled by α-secretase mediated non-amyloidogenic pathway and β, γ-secretase-mediated amyloidogenic pathway [22]. However, APP levels are not different between the two genotype littermates, suggesting that AQP4 deficiency does not affect Aβ production. Also, AQP4 deficiency does not alter expression levels of proteins associated with Aβ formation and degradation. Therefore, we believe that increased Aβ load in AQP4^−/−^APP/PS1 brain is due to more severe impairment of Aβ clearance, via the following mechanisms.

First, the lack of AQP4 mediated paravascular pathways of Aβ clearance could be a critical factor for heightened Aβ accumulation in AQP4^−/−^APP/PS1 brain. It is known that AQP4 mediated rapid transport of intercellular water serves as a main “driving force” for clearance of interstitial solutes, including Aβ, from the brain parenchyma into the blood and CSF [3–6]. Deletion of the AQP4 gene damages rapid water transport-dependent clearance of ^125^I-Aβ_1–40_ from brain parenchyma to the blood and CSF [5]. In the present study, AQP4^−/−^APP/PS1 mice show increases in amyloid plaque deposit in the hippocampus and cerebral cortex and high concentrations of Aβ_1–40_ and Aβ_1–42_ in brain protein extraction, compared with APP/PS1 transgenic mice. AQP4 deficiency in APP/PS1 mouse brain improving amyloid load within the walls of brain vessels and surfaces of pia mater further supports a facilitating role of AQP4 in the perivascular glymphatic drainage of Aβ.

Astrocyte atrophy, with attenuation of reactive gliosis and low expression of LRP1, may be another critical factor for heightened Aβ accumulation in AQP4^−/−^APP/PS1 brain. Reactive astrogliosis surrounding Αβ plaques has been shown to limit plaque growth. Attenuating astrocyte activation, by deletions of GFAP and vimentin, accelerates amyloid plaque load in APP/PS1 mice [13]. By contrast, enhancing astrocyte lysosome biogenesis with transcription factor EB, a master regulator of lysosome biogenesis, facilitates Aβ clearance in APP/PS1 mice [25]. The present pathological analyses have shown that reactive hypertrophic astrocytes, with up-regulation of LRP1 and AQP4, surround Aβ plaques in 12-month old APP/PS1 mice. This result is consistent with our previous studies showing that activated cultured astrocytes increase both LRP1 and AQP4 expression in response to Aβ treatment [18]. LRP1 is shown to mediate clearance mechanisms of brain Aβ, via either its uptake into brain cells with subsequent degradation or its transport across the blood–brain-barrier into the periphery [33]. Therefore, reactive astrogliosis may perform a plaque-clearance effect via LRP1 binding Aβ, thereby decreasing the concentration of Aβ in the extracellular space, and subsequently attenuating Aβ toxicity. However, AQP4 deficiency alters these effects of reactive astrocytes, exacerbating Aβ plaque deposit and soluble Aβ accumulation. Increased Aβ load improves its toxicity on astrocytes, resulting in astrocyte atrophy with down-regulation of LRP1. This, in turn, affects Aβ clearance, thus forming a vicious cycle between Aβ accumulation and astrocyte damage in the AD-like pathology.

The present results also reveal that genetic deletion of AQP4 has a tendency to reduce the number of microglia surrounding Aβ plaques in 12 month-old APP/PS1 mice. Serving as macrophage-like cells in the CNS, undoubtedly activated microglia perform a scavenging effect on Aβ plaque and degenerated neuronal components [31]. Mildly reduced microgliosis may also be a contributor to increased Aβ load in AQP4^−/−^APP/PS1 mouse brain. The attenuation of microglia activation could be a consequence of reduced astrogliosis, since microglia do not express AQP4. In the acute stages of neurotrauma, stroke and neuroinflammation, microglia are activated and subsequently evoke astrocyte activation via the release of pro-inflammatory cytokines and chemokines. In the chronic stages, activated astrocytes also produce similar cytokines for self-regulation and for the regulation of microglia [52]. This phenomenon creates a feedback loop, allowing both microglia and astrocytes to regulate one another. In agreement with the present finding, reduced reactive astrogliosis and microgliosis is also observed in AQP4^−/−^ mice in experimental autoimmune encephalomyelitis and after intracerebral injection of LPS [19, 20]. AQP4 deletion impairs glial scar formation and reduces neuroinflammation in the acute stage of post-traumatic impairment, but improves neurodegeneration and reactive gliosis in the chronic stage, which could possibly be attributed to prolonged impairment of extracellular tau clearance along glymphatic paravascular pathways [3]. Our studies also show that AQP4 deletion in APP/PS1 mice improves reactive astrogliosis at age of 6.5 months, but attenuates astrogliosis at 12 months, which could be due to astrocyte atrophy caused by increased Aβ accumulation. Together, these studies suggest the effect of AQP4 deletion on astrocyte reactivity depends on the types and stages of neurological diseases.

Additionally, previous studies have indicated that AQP4 has an intrinsic proinflammatory role during astrocyte activation [20]. This view is further supported by reduced cytokine (IL-6 and TNF-α) secretion in AQP4^−/−^ astrocyte cultures exposed to LPS [19]. The present results also indicate that AQP4 knockout has a tendency to attenuate glial inflammation in 12 month-old APP/PS1 brain, reflected by significant decreases in IL-1β and nonsignficant decreases in IL-6 and TNF-α in hippocampal and cerebral samples. Compared with IL-6, IL-1β is produced by reactive astrocytes already in a very early stage of Aβ deposition [52, 53]. Thus, reduced reactive astrocytes could possibly be a key factor for ameliorating IL-1β increases in AQP4^−/−^ APP/PS1 brain. Aside from reactive astrocytes and microglia, IL-6 and TNF-α are also produced by degenerative neurons [54, 55]. Thus, more severe neurodegeneration in AQP4^−/−^APP/PS1 mice may increase neuronal IL-6 and TNF-α expression, leading to a compensatory role for reduced IL-6 and TNF-α produced by reactive glia. Although the long term deletion of AQP4 has a tendency to attenuate the glial inflammation that has detrimental effects on the brain, it should be noted that it impairs beneficial effects of reactive gliosis, such as up-regulation of BDNF expression [56], facilitating Aβ clearance and restricting the growth of Aβ plaques [13, 25, 56]. This further exacerbates synaptic and cognitive impairments in APP/PS1 mice.

Activated astrocytes with overexpression of AQP4 in the vicinity of amyloid plaque may facilitate Aβ removal and limit plaque growth in APP/PS1 mice. Nevertheless, loss of perivascular AQP4 polarization would impair perivascular clearance of Aβ, causing amyloid deposition within the walls of cortical and pial vessels [7, 57]. AQP4 is highly expressed in astrocyte processes abutting cerebral microvessels or pia, while its density is comparatively low at astrocyte membrane facing neuropil regions [8, 40]. Several studies have suggested that AQP4 mediating drainage of ISF and solutes from brain parenchyma along vascular walls are dependent on the perivascular AQP4 polarization [5, 12]. In deed, loss of perivascular AQP4 polarization coincides with impaired clearance of exogenous Aβ and Tau from the cerebral cortex [3, 6]. In the present study, we found that the polarized expression of AQP4 in perivascular astrocyte endfeet, as well as glial limitans along the cortical surface, was progressively disrupted during the neuropathological progress of APP/PS1 mice. This was mainly due to widespread astrogliosis with marked upregulation of AQP4 in the neuropil, especially those surrounding plaques. Although we have demonstrate that deletion of AQP4 exacerbates CAA, further studies are necessary to indentify the exact causal relationship between impaired perivascular AQP4 polarization and vascular Aβ deposits in APP/PS1 mice at the different ages. It is also necessary to investigate the effect of AQP4 deletion on the onset of early AD-related cognitive deficit and Aβ pathogenesis in APP/PS1 mice at younger age in addition to 12 months of age. In addition, it has been shown that paravascular Aβ clearance is regulated by the sleep-wake cycle, which is disturbed in many patients with AD [58]. It will be interesting to investigate whether deletion of AQP4 may affect this pathway in both physiological and pathological conditions.

## Conclusions

In summary, the present study demonstrates that AQP4 attenuates brain Aβ accumulation and memory deficits in APP/PS1 mice. AQP4 mediated clearance of soluble Aβ from the brain parenchyma in AD pathology may occur via two main mechanisms: the paravascular pathway and glial activation. It should be further addressed the roles played by each in the various stages of AD. Given the potentially proinflammatory features of AQP4, effective dosage and treatment time-window of AQP4 receptor agonists in the treatment of AD need further evaluation. Establishing whether its inflammatory side-effects can be reduced by combined treatment with non-steroidal anti-inflammatory drugs also needs further investigation. Exploring these issues will be conducive to providing theoretical and experimental basis for clinical therapy of AD via targeting AQP4.

## Methods

### Ethics statement

Animal experiments were performed in accordance with the recommendations in the Guide for the Care and Use of Laboratory Animals of Nanjing Medical University. All of the animals were handled according to approved by the Institutional Animal Care and Use Committee (NJMU) protocol 1210.

### Mice

APP695/PS1-dE9 transgenic (APP/PS1) mice with a C57BL/6 J background were obtained from Jackson Laboratories. AQP4^−/−^ mice in a CD1 genetic background have been established in our laboratory [59] and successfully used for investigating roles of AQP4 in astrocyte function and neuropsychiatric disorders [15–18, 21, 46, 48]. Adult APP/PS1 mice were crossed with female AQP4^−/−^ mice to produce AQP4^+/−^APP/PS1 mice. Female and male AQP4^+/−^APP/PS1 mice were crossed to produce AQP4^+/−^APP/PS1 and AQP4^+/−^APP/PS1^−/−^ offspring, which were then mated to generate AQP4^+/+^APP/PS1^−/−^ (WT) mice, AQP4^+/+^APP/PS1 (also named APP/PS1) mice, AQP4^−/−^APP/PS1^−/−^ (also named AQP4^−/−^) mice and AQP4^−/−^APP/PS1 mice that are all of the same mixed background (C57BL/6 J CD1). The mouse genotype was identified using polymerase chain reaction, as previously described [59, 60] (Additional file 4: Figure S4). Following weaning, same-sex littermates were housed 3–4 per cage, until they were 11.5-12 month-old. One mouse per cage was then randomly selected for behavioral testing. A subgroup of APP/PS1 mice and AQP4^−/−^APP/PS1^−/−^ mice at 6.5 months was used for analyses of reactive astrogliosis and Aβ plaque deposit. Several WT and APP/PS1 mice at 3, 6.5 and 12 months of age were used for analysis AQP4 polarization.

### Y-maze test

The Y-maze test was conducted to evaluate the short-term spatial working memory of the mice, as previously described [61]. The test contains two, 5-min stages with an interval of 2 h between evaluation periods. During the first stage, the novel arm was blocked by a black baffle, allowing mice to only move in the other two arms. During the second stage, the novel arm was open, and mice could freely move throughout the three arms. The percentage of time traveled in each arm, number of entries into each arm, and travelling speed was calculated.

### Morris water maze

The Morris water maze task was performed to measure long-term spatial learning and memory function, as described previously [16]. Training was conducted over 8 consecutive days, with 4 trials per day. During the first two days, mice were trained to find a dark-colored cylindrical platform with a diameter of 10 cm, sitting 0.5 cm above the water surface. Mice were not given the next hidden platform tests if they had apparent motor and/or visual deficits indicated by long escape latency (>50 s) and low swimming speed (<75 mm/s). On the 3^rd^ day, the platform was moved to the opposite quadrant and submerged 1 cm below the surface of the water. The escape latency, swimming distance and swimming speed were analyzed. On day 9, the hidden platform was removed, allowing mice to swim in the pool for 60 s. The percentage of total time spent in each quadrant, and the number of crossing where the platform had been previously located, were analyzed.

Mouse activity in the aforementioned behavioral apparatuses was collected by a digital video camera connected to a computer-controlled system (Beijing Sunny Instruments Co. Ltd, China). All tests were performed by two independent experimenters, who were each blind to the treatment schedule.

### Section and tissue preparation

After anesthesia, mice were transcardially perfused with 0.9 % saline, followed by 4 % paraformaldehyde by perfusion pump (Cole-parmer, USA). Brains were dissected mid-sagittallly, postfixed overnight at 4 °C, dehydrated in a series of graded ethanol solutions then embedded in paraffin. Sagittal brain sections were serially cut at 5 μm using a paraffin slicing machine (Leica RM2135, Nussloch, Germany), and collected form Lateral −0.96 to −1.92 mm in the mouse brain atlas as landmarks [62]. Every tenth section was collected as 1 set, and 10 sets per brain hemisphere were obtained. For biochemical analyses, mice were euthanized by decapitation. The cerebral cortex and hippocampus were promptly dissected. Tissues were flash frozen in liquid nitrogen and stored at −80 °C until analysis.

### Immunohistochemistry

Immunohistochemical staining was performed as previously described [16]. Briefly, one set of brain sections per mouse were respectively incubated with one of the following primary antibodies: rabbit anti-6E10 (1:1000; Covance, Princeton, NJ, USA), mouse anti-GFAP (1:1000; Millipore, Billerica, MA, USA), mouse anti-Iba-1 (1:500; Wako, Osaka, Japan), mouse anti-SYP (1:1000; Millipore), rabbit anti-PSD-95 (1:500; Abcam, Cambridge, MA, USA), rabbit anti-LRP1 (1:200; Santa Cruz Biotechnology, Santa Cruz, CA, USA) or rabbit anti-AQP4 (1:500; Millipore) overnight at 4 °C. Following PBS rinsing, the sections were incubated with biotinylated goat anti-mouse or rabbit IgG (1:400) for 1 h at room temperature and thereafter incubated with streptavidin-biotin-peroxidase complex (Elite ABC Kit, Vector Laboratories, Burlingame, CA, USA). All experimental conditions including incubation time of 3,3′-diaminobenzidine (DAB) staining were kept in consistence with each other. In general the antibody-bound peroxidase was visualized by incubating sections for 3–4 min in 0.05 % DAB with H_2_O_2_ to avoid that staining intensity could be saturated. Sections with GFAP, Iba-1 or AQP4 immunostaining were counterstained by Congo Red.

Immunohistochemical controls were taken by omitting the primary antibodies or replacing with an equivalent concentration of either normal mouse serum or normal rabbit serum. The immunostaining for AQP4 was also performed on brain sections of AQP4^−/−^ mice to exam the specificity of the antibody. All sections were immuno-negative.

### Immunofluorescence

Paraffin-embedded tissue sections were deparaffinized and hydrated through a series of graded ethanol solutions, followed by 0.1 M Tris, pH 7.6. Slices were blocked for 1 h at room temperature with 3 % normal goat serum, incubated with primary antibodies including mouse anti-GFAP (1:500, Millipore) and rabbit anti-LRP1 (1:200; Santa Cruz Biotechnology) overnight at 4 °C. After extensive rinsing, sections were incubated for 2 h at room temperature in a mixture of FITC-conjugated goat anti-mouse IgG (1:150, Vector laboratories) and Texas Red-conjugated goat anti-rabbit IgG (1:150, Vector Laboratories). The sections were washed for 3 × 5 min in PBS containing 1.5 μM 4',6-diamidino-2-phenylindole (DAPI, Invitrogen, Carlsbad, CA, USA) and then cover-slipped with buffered PBS/glycerol.

### ELISA Analysis

Cerebral cortex and hippocampus samples were homogenized and sonicated in ice-cold TBS buffer containing 0.5 mM PMSF, 0.5 mM benzamidine, 1.0 mM DTT and 1.0 mM EDTA, followed by centrifugation at 100,000 × g for 1 h. Supernatants were set aside for measurements utilizing soluble Aβ_1–40_ and Aβ_1–42_, IL-1β, IL-6, TNF-α and BDNF. Pellets were re-suspended and further homogenized in 70 % formic acid (equal volume of TBS), then centrifuged at 100,000 × g for 1 h. Formic acid supernatants were neutralized with 1 M Tris for insoluble Aβ_1–40_ and Aβ_1–42_ analysis [63]. The above indexes were quantified with ELISA kits from Invitrogen Corporation according the manufacturer’s instructions.

### Western blot

Cerebral cortex and hippocampus extracts were loaded onto 10-16 % Tris/tricine SDS gels, and transferred to nitrocellulose membranes before overnight incubation with one of the following primary antibodies: rabbit anti-APP (1:1000; Millipore), rabbit anti-Aβ_1–42_ (1:1000; Sigma-Aldrich, Saint Louis, MO, USA) and mouse anti-SAPPβ (1:1000; Sigma-Aldrich), mouse anti-CTFβ (1:1500; Millipore), mouse anti-BACE1 (1:2000; Millipore), rabbit anti-PS1 (1:1000; Sigma-Aldrich), rabbit anti-NEP (1:1000; Millipore), rabbit anti-IDE (1:1000; Abcam, Cambridge, MA, USA), mouse anti-GFAP (1:2000; Millipore), mouse anti-Iba-1 (1:500; Wako), mouse anti-SYP (1:1500; Millipore), rabbit anti-PSD-95 (1:1000; Abcam), mouse anti-BDNF (1:500; Sigma-Aldrich) or rabbit anti-β-tubulin (1:3000; Sigma-Aldrich). Horseradish peroxidase-conjugated secondary antibodies (Vector Laboratories) were used, and bands were visualized using ECL plus detection system. β-tubulin was utilized as an internal control for protein loading and transfer efficiency.

### Quantitative analyses of plaque load and immunohistochemistry

Sections were visualized using a digital microscope (Leica Microsystems, Wetzlar, Germany) and captured with constant exposure time, offset, and gain for each staining marker. For analysis of the percentage area of positive signal for Thioflavin-S, 6E10, GFAP, Iba-1, LRP1, SYP or PSD-95, the corresponding images of the hippocampus, together with the cerebral cortex immediately dorsal to the hippocampus, were captured at × 100 magnification, and exported to Image-Pro Plus 6.0 Analysis System (Media Cybernetics Inc., San Francisco, CA, USA). The cerebral and hippocampal areas in each section were manually delineated. The area of positive signal was measured using the interest grayscale threshold analysis with constant settings for minimum and maximum intensities for each staining marker as described previously [64]. The percentage area of positive signal was calculated by dividing the area of positive signal to the total area in the region of interest.

For analysis of 6E10 expression on the CAA, cross-sectional areas of GFAP or Iba-1 positive cells around plaques, LRP1 expression around plaques, or AQP4 polarization, two images at 400× magnification were randomly captured from the outer layers of the cortex and the lacunosum moleculare layer of the hippocampus on each brain section, respectively. The mean 6E10-IR intensity along the walls of cortical and leptomeningeal vessels was measured by Image-Pro Plus Software. The cell surface area of GFAP-positive astrocytes or Iba-1 positive microglia with somata within 100 μm from the plaque border was also measured. For analysis of AQP4 expression and localization, the mean AQP4-IR intensity at the regions immediately abutting large vessels (diameter > 25 μm), microvessels (diameter < 10 μm) or pia maters and correspondingly adjacent parenchymal domains was measured. Perivascular (or pia) AQP4 polarization was obtained by comparing expression ratios of AQP4 at perivascular (or pia surface) versus parenchymal domains [6, 41]. For analysis of plaques related LRP1 expression, the percentage of LRP1 positive area within a radius of 100 μm to the plaque border was measured on the immunofluorescent sections. The percentage of LRP1 and GFAP double positive area and the percentage of LRP1 positive and GFAP negative area were also calculated.

Eight-nine brain sections in each set were averaged for each mouse, and 5–6 mice were averaged for each genotype group. All quantification was done blind to animal genotype.

### Statistical analysis

All data were expressed as means ± SEM. Using SPSS software, version 16.0 (SPSS Inc., USA), data for the Morris water maze platform training were analyzed by repeated-measures analysis of variance (ANOVA) followed by Newman-Keuls post-hoc multiple comparison test. Other data were analyzed by ANOVA, followed by Newman-Keuls post-hoc multiple comparison test or Student’s *t*-test as indicated in the figure legends.

## Additional files

Additional file 1: Figure S1. The examples of several antibodies including APP, BACE1, PS1, and IDE, NEP and β-tubulin on the whole membranes, and found that all these proteins were detected at the position of the corresponding molecular weight (indicated by arrowheads). (TIF 1697 kb) Additional file 2: Figure S2. AQP4 deficiency increased reactive astrogliosis and Aβ plaque deposits in 6.5 month-old APP/PS1 mice. (A) GFAP immunostaining counterstained with Congo Red. AQP4^−/−^APP/PS1 mice had more extensive reactive astrogliosis in the cerebral cortex and hippocampus than APP/PS1controls. (B) Thioflavin-S staining showing Aβ load in the cortex and hippocampus. (C) The percentage area of GFAP positive. (D) The percentage area of thioflavin-S positive. Data represent mean ± SEM from 5 mice (3 female, and 2 male) per group and analyzed by Student’s *t*-test. **P* < 0.05. (TIF 4322 kb) Additional file 3: Figure S3. AQP4 deficiency did not affect LRP1 expression by neurons in APP/PS1 mice. (A) LRP1 immunohistochemistry. High expression of LRP1 was localized to the cell membrane of pyramidal neurons in 12-month old AQP4^−/−^APP/PS1 mice and APP/PS1 mice. Some glial-like cells (inserted high magnification images) distal to the plaques were also positive for LRP1 in the two genotype mice. (C) Quantitative analysis of the percentage of LRP-1 positive area in the hippocampus and cerebral cortex. (B) Western bolt and (D) densitometry analysis of LRP1 protein levels in the hippocampus and cortex. Data represent mean ± SEM from 5–6 mice (3–4 female, and 1–2 male) per group. The statistical analysis was performed by Student’s *t*-test. (TIF 3855 kb) Additional file 4: Figure S4. Genotyping of mice. An example of PCR analysis of AQP4 (the upper panel) and APPSwe (the low panel) mRNA expression in a newborn litter that is generated by AQP4^+/−^APP/PS1^+/+^ and AQP4^+/−^APP/PS1^−/−^ mice. We only detected APPSwe transgene, because APPSwe transgene and PS1ΔE9 transgene are coexpressed under the control of the mouse prion promoter (Jankowsky et al. [60]). APP/PS1 transgene allele yields a 377-bp product; and wild type allele has no product. AQP4 knockout homozygote allele yields a 320-bp product; heterozygote allele yields 240-bp and 320-bp products; and wild type allele yields a 240-bp product. In this litter, eleven mice belong to the following 6 genotypes: AQP4^+/+^APP/PS1^+/+^ (mice 1 and 4), AQP4^+/−^APP/PS1^−/−^ (mouse 2), AQP4^+/−^APP/PS1^+/+^ (mice 3, 8 and 9), AQP4^−/−^APP/PS1^+/+^ (mice 5 and 6), AQP4^−/−^APP/PS1^−/−^ (mice 7 and 10), and AQP4^+/+^APP/PS1^−/−^ (mouse 11). (TIF 2043 kb)

## Abbreviations

Aβ: amyloid-beta
AD: Alzheimer’s disease
ANOVA: analysis of variance
APP: amyloid-beta precursor protein
AQP4: aquaporin-4
BACE1: β-site amyloid precursor protein-cleaving enzyme 1
BDNF: brain-derived neurotrophic factor
CAA: cerebral amyloid angiopathy
CTFβ: transmembrane peptide C-terminal fragment
GFAP: glial fibrillary acidic protein
Iba-1: Ionized calcium-binding adaptor molecule-1
IDE: insulin degrading enzyme
IL-1β: interleukin-1 beta
IL-6: interleukin-6
IR: immunoreactive
ISF: interstitial solutes
LRP1: low density lipoprotein receptor-related protein-1
NEP: neprilysin
PS1: presenilin 1
PSD-95: Post-synaptic density protein-95
SAPPβ: soluble peptide APP β
SYP: synaptophysin
TNF-α: tumor necrosis factor alpha

## References

[1] Huang Y, Mucke L (2012). Alzheimer mechanisms and therapeutic strategies. Cell.

[2] Shankar GM, Walsh DM (2009). Alzheimer’s disease: synaptic dysfunction and Abeta. Mol Neurodegener.

[3] Iliff JJ, Chen MJ, Plog BA, Zeppenfeld DM, Soltero M, Yang L (2014). Impairment of glymphatic pathway function promotes tau pathology after traumatic brain injury. J Neurosci.

[4] Iliff JJ, Lee H, Yu M, Feng T, Logan J, Nedergaard M (2013). Brain-wide pathway for waste clearance captured by contrast-enhanced MRI. J Clin Invest.

[5] Iliff JJ, Wang M, Liao Y, Plogg BA, Peng W, Gundersen GA (2012). A paravascular pathway facilitates CSF flow through the brain parenchyma and the clearance of interstitial solutes, including amyloid β. Sci Transl Med.

[6] Kress BT, Iliff JJ, Xia M, Wang M, Wei HS, Zeppenfeld D (2014). Impairment of paravascular clearance pathways in the aging brain. Ann Neurol.

[7] Arbel-Ornath M, Hudry E, Eikermann-Haerter K, Hou S, Gregory JL, Zhao L (2013). Interstitial fluid drainage is impaired in ischemic stroke and Alzheimer’s disease mouse models. Acta Neuropathol.

[8] Papadopoulos MC, Verkman AS (2013). Aquaporin water channels in the nervous system. Nature Rev Neurosci.

[9] Hoshi A, Yamamoto T (2012). Characteristics of aquaporin expression surrounding senile plaques and cerebral amyloid angiopathy in Alzheimer disease. J Neuropathol Exp Neurol.

[10] Moftakhar P, Lynch MD, Pomakian JL, Vinters HV (2010). Aquaporin expression in the brains of patients with or without cerebral amyloid angiopathy. J Neuropathol Exp Neurol.

[11] Pérez E, Barrachina M, Rodríguez A, Torrejón-Escribano B, Boada M, Hernández I (2007). Aquaporin expression in the cerebral cortex is increased at early stages of Alzheimer disease. Brain Res.

[12] Yang J, Lunde LK, Nuntagij P, Oguchi T, Camassa LM, Nilsson LN (2011). Loss of astrocyte polarization in the tg-ArcSwe mouse model of Alzheimer’s disease. J Alzheimers Dis.

[13] Kraft AW, Hu X, Yoon H, Yan P, Xiao Q, Wang Y (2013). Attenuating astrocyte activation accelerates plaque pathogenesis in APP/PS1 mice. FASEB J.

[14] Auguste KI, Jin S, Uchida K, Yan D, Manley GT, Papadopoulos MC (2007). Greatly impaired migration of implanted aquaporin-4-deficient astroglial cells in mouse brain toward a site of injury. FASEB J.

[15] Fan Y, Kong H, Shi X, Sun X, Ding J, Wu J (2008). Hypersensitivity of aquaporin 4-deficient mice to 1-methyl-4-phenyl-1,2,3,6-tetrahydropyrindine and astrocytic modulation. Neurobiol Aging.

[16] Liu L, Lu Y, Kong H, Li L, Marshall C, Xiao M (2012). Aquaporin-4 deficiency exacerbates brain oxidative damage and memory deficits induced by long-term ovarian hormone deprivation and D-galactose injection. Int J Neuropsychopharmacol.

[17] Wu Q, Zhang YJ, Gao JY, Li XM, Kong H, Zhang YP (2014). Aquaporin-4 mitigates retrograde degeneration of rubrospinal neurons by facilitating edema clearance and glial scar formation after spinal cord injury in mice. Mol Neurobiol.

[18] Yang W, Wu Q, Yuan C, Gao J, Xiao M, Gu M (2012). Aquaporin-4 mediates astrocyte response to β-amyloid. Mol Cell Neurosci.

[19] Li L, Zhang H, Varrin-Doyer M, Zamvil SS, Verkman AS (2011). Proinflammatory role of aquaporin-4 in autoimmune neuroinflammation. FASEB J.

[20] Li L, Zhang H, Verkman AS (2009). Greatly attenuated experimental autoimmune encephalomyelitis in aquaporin-4 knockout mice. BMC Neurosci.

[21] Fan Y, Liu M, Wu X, Wang F, Ding J, Chen J (2013). Aquaporin-4 promotes memory consolidation in Morris water maze. Brain Struct Funct.

[22] Tanzi RE, Bertram L (2005). Twenty years of the Alzheimer’s disease amyloid hypothesis: a genetic perspective. Cell.

[23] Eckman EA, Eckman CB (2005). Abeta-degrading enzymes: modulators of Alzheimer’s disease pathogenesis and targets for therapeutic intervention. Biochem Soc Trans.

[24] Takata K, Kitamura Y, Yanagisawa D, Morikawa S, Morita M, Inubushi T (2007). Microglial transplantation increases amyloid-beta clearance in Alzheimer model rats. FEBS Lett.

[25] Xiao Q, Yan P, Ma X, Liu H, Perez R, Zhu A (2014). Enhancing astrocytic lysosome biogenesis facilitates Aβ clearance and attenuates amyloid plaque pathogenesis. J Neurosci.

[26] Kobayashi K, Hayashi M, Nakano H, Shimazaki M, Sugimori K, Koshino Y (2004). Correlation between astrocyte apoptosis and Alzheimer changes in gray matter lesions in Alzheimer’s disease. J Alzheimers Dis.

[27] Takuma K, Baba A, Matsuda T (2004). Astrocyte apoptosis: implications for neuroprotection. Prog Neurobiol.

[28] Kulijewicz-Nawrot M, Verkhratsky A, Chvátal A, Syková E, Rodríguez JJ (2012). Astrocytic cytoskeletal atrophy in the medial prefrontal cortex of a triple transgenic mouse model of Alzheimer’s disease. J Anat.

[29] Olabarria M, Noristani HN, Verkhratsky A, Rodríguez JJ (2010). Concomitant astroglial atrophy and astrogliosis in a triple transgenic animal model of Alzheimer’s disease. Glia.

[30] Fuller S, Steele M, Münch G (2010). Activated astroglia during chronic inflammation in Alzheimer’s disease--do they neglect their neurosupportive roles?. Mutat Res.

[31] Schlachetzki JC, Hüll M (2009). Microglial activation in Alzheimer’s disease. Curr Alzheimer Res.

[32] Deane R, Wu Z, Zlokovic BV (2004). RAGE (yin) versus LRP (yang) balance regulates alzheimer amyloid beta-peptide clearance through transport across the blood–brain barrier. Stroke.

[33] Jaeger S, Pietrzik CU (2008). Functional role of lipoprotein receptors in Alzheimer’s disease. Curr Alzheimer Res.

[34] Savioz A, Leuba G, Vallet PG (2014). A framework to understand the variations of PSD-95 expression in brain aging and in Alzheimer’s disease. Ageing Res Rev.

[35] Shao CY, Mirra SS, Sait HB, Sacktor TC, Sigurdsson EM (2011). Postsynaptic degeneration as revealed by PSD-95 reduction occurs after advanced Aβ and tau pathology in transgenic mouse models of Alzheimer’s disease. Acta Neuropathol.

[36] Sze CI, Troncoso JC, Kawas C, Mouton P, Price DL, Martin LJ (1997). Loss of the presynaptic vesicle protein synaptophysin in hippocampus correlates with cognitive decline in Alzheimer disease. J Neuropathol Exp Neurol.

[37] Monteggia LM, Barrot M, Powell CM, Berton O, Galanis V, Gemelli T (2004). Essential role of brain-derived neurotrophic factor in adult hippocampal function. Proc Natl Acad Sci U S A.

[38] Tong L, Balazs R, Thornton PL, Cotman CW (2004). Beta-amyloid peptide at sublethal concentrations downregulates brain-derived neurotrophic factor functions in cultured cortical neurons. J Neurosci.

[39] Nagahara AH, Merrill DA, Coppola G, Tsukada S, Schroeder BE, Shaked GM (2009). Neuroprotective effects of brain-derived neurotrophic factor in rodent and primate models of Alzheimer’s disease. Nat Med.

[40] Nagelhus EA, Ottersen OP (2013). Physiological roles of aquaporin-4 in brain. Physiol Rev.

[41] Wang M, Iliff JJ, Liao Y, Chen MJ, Shinseki MS, Venkataraman A (2012). Cognitive deficits and delayed neuronal loss in a mouse model of multiple microinfarcts. J Neurosci.

[42] Mawuenyega KG, Sigurdson W, Ovod V, Munsell L, Kasten T, Morris JC, Yarasheski KE, Bateman RJ. Decreased clearance of CNS beta-amyloid in Alzheimer's disease. Science. 2010;330:1774. 10.1126/science.1197623 PMC3073454 21148344

[43] Schnabel J (2011). Amyloid: little proteins, big clues. Nature.

[44] Xiao M, Hu G (2014). Involvement of aquaporin 4 in astrocyte function and neuropsychiatric disorders. CNS Neurosci Ther.

[45] Skucas VA, Mathews IB, Yang J, Cheng Q, Treister A, Duffy AM (2011). Impairment of select forms of spatial memory and neurotrophin-dependent synaptic plasticity by deletion of glial aquaporin-4. J Neurosci.

[46] Zhang J, Li Y, Chen ZG, Dang H, Ding JH, Fan Y (2013). Glia protein aquaporin-4 regulates aversive motivation of spatial memory in Morris water maze. CNS Neurosci Ther.

[47] Zador Z, Stiver S, Wang V, Manley GT (2009). Role of aquaporin-4 in cerebral edema and stroke. Handb Exp Pharmacol.

[48] Tang Y, Wu P, Su J, Xiang J, Cai D, Dong Q (2010). Effects of Aquaporin-4 on edema formation following intracerebral hemorrhage. Exp Neurol.

[49] Papadopoulos MC, Verkman AS (2012). Aquaporin 4 and neuromyelitis optica. Lancet Neurol.

[50] Binder DK, Nagelhus EA, Ottersen OP (2012). Aquaporin-4 and epilepsy. Glia.

[51] Benilova I, Karran E, De Strooper B (2012). The toxic Aβ oligomer and Alzheimer’s disease: an emperor in need of clothes. Nat Neurosci.

[52] Apelt J, Schliebs R (2001). β-Amyloid-induced glial expression of both pro- and anti-inflammatory cytokines in cerebral cortex of aged transgenic Tg2576 mice with Alzheimer plaque pathology. Brain Res.

[53] Mehlhorn G, Hollborn M, Schliebs R (2000). Induction of cytokines in glial cells surrounding cortical b-amyloid plaques in transgenic Tg2576 mice with Alzheimer pathology. Int J Dev Neurosci.

[54] Erta M, Quintana A, Hidalgo J (2012). Interleukin-6, a major cytokine in the central nervous system. Int J Biol Sci.

[55] Vitkovic L, Konsman JP, Bockaert J, Dantzer R, Homburger V, Jacque C (2000). Cytokine signals propagate through the brain. Mol Psychiatry.

[56] Kimura N, Takahashi M, Tashiro T, Terao K (2006). Amyloid beta up-regulates brain-derived neurotrophic factor production from astrocytes: rescue from amyloid beta-related neuritic degeneration. J Neurosci Res.

[57] Hawkes CA, Jayakody N, Johnston DA, Bechmann I, Carare RO (2014). Failure of perivascular drainage of β-amyloid in cerebral amyloid angiopathy. Brain Pathol.

[58] Xie L, Kang H, Xu Q, Chen MJ, Liao Y, Thiyagarajan M (2013). Sleep drives metabolite clearance from the adult brain. Science.

[59] Fan Y, Zhang J, Sun XL, Gao L, Zeng XN, Ding JH (2005). Sex- and region-specific alterations of basal amino acid and monoamine metabolism in the brain of aquaporin-4 knockout mice. J Neurosci Res.

[60] Jankowsky JL, Slunt HH, Ratovitski T, Jenkins NA, Copeland NG, Borchelt DR (2001). Co-expression of multiple transgenes in mouse CNS: a comparison of strategies. Biomol Eng.

[61] Wang Q, Xu Z, Tang J, Sun J, Gao J, Wu T (2005). Voluntary exercise counteracts Aβ25-35-induced memory impairment in mice. Behav Brain Res.

[62] Franklin KBJ, Paxinos G (2008). The mouse brain in stereotaxic coordinates.

[63] Suh J, Choi SH, Romano DM, Gannon MA, Lesinski AN, Kim DY (2013). ADAM10 missense mutations potentiate β-amyloid accumulation by impairing prodomain chaperone function. Neuron.

[64] Kim J, Castellano JM, Jiang H, Basak JM, Parsadanian M, Pham V (2009). Overexpression of low-density lipoprotein receptor in the brain markedly inhibits amyloid deposition and increases extracellular A beta clearance. Neuron.

