# Supplementary figures and images for: Deletion of aquaporin-4 in APP/PS1 mice exacerbates brain Aβ accumulation and memory deficits
Source: Mol Neurodegener. 2015 Nov 2;10:58. doi: 10.1186/s13024-015-0056-1 (PMC4631089; doi:10.1186/s13024-015-0056-1)

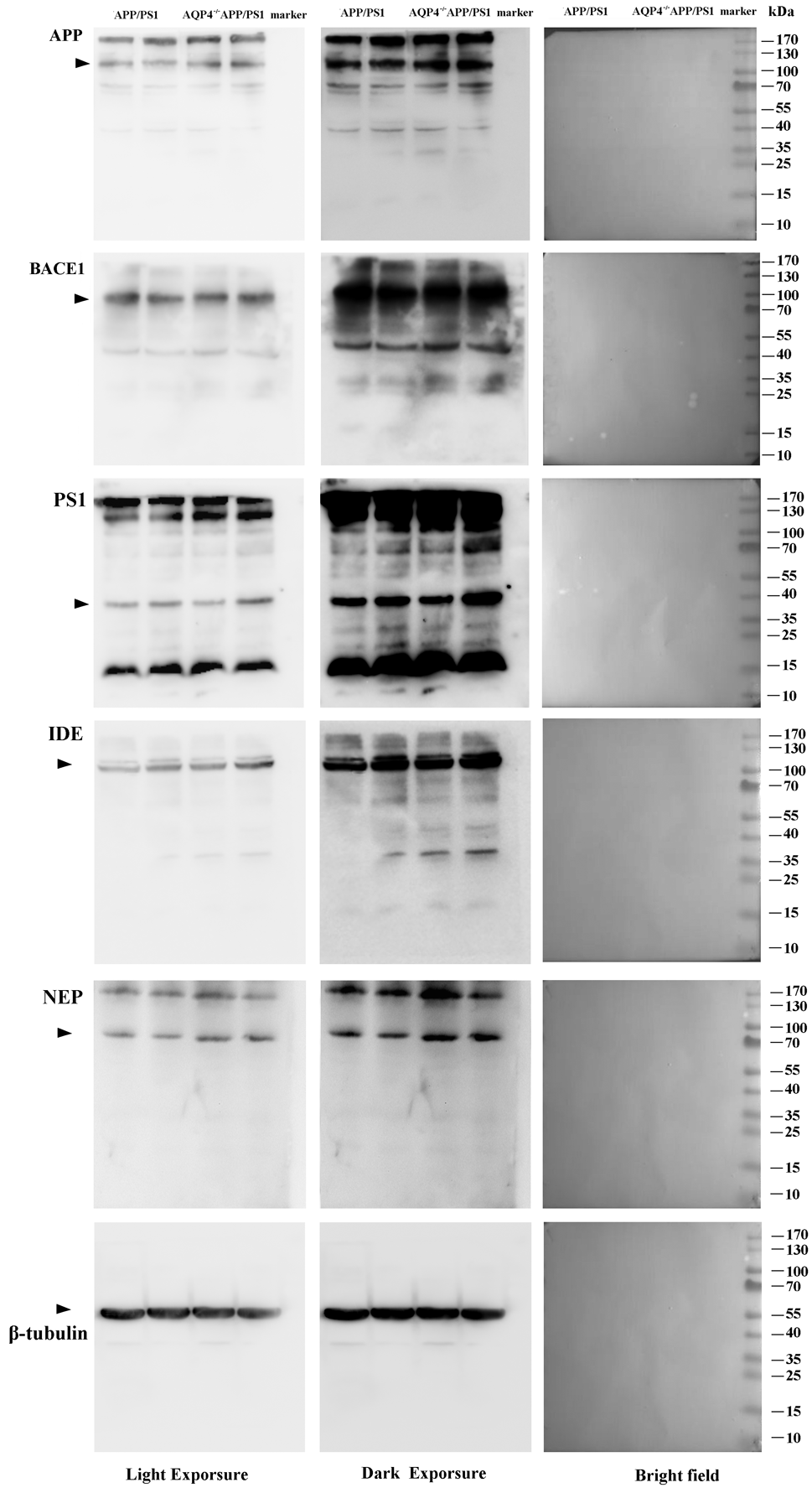

Supplement: Additional file 1: Figure S1. — The examples of several antibodies including APP, BACE1, PS1, and IDE, NEP and β-tubulin on the whole membranes, and found that all these proteins were detected at the position of the corresponding molecular weight (indicated by arrowheads). (TIF 1697 kb) [file 13024_2015_56_MOESM1_ESM.tif]

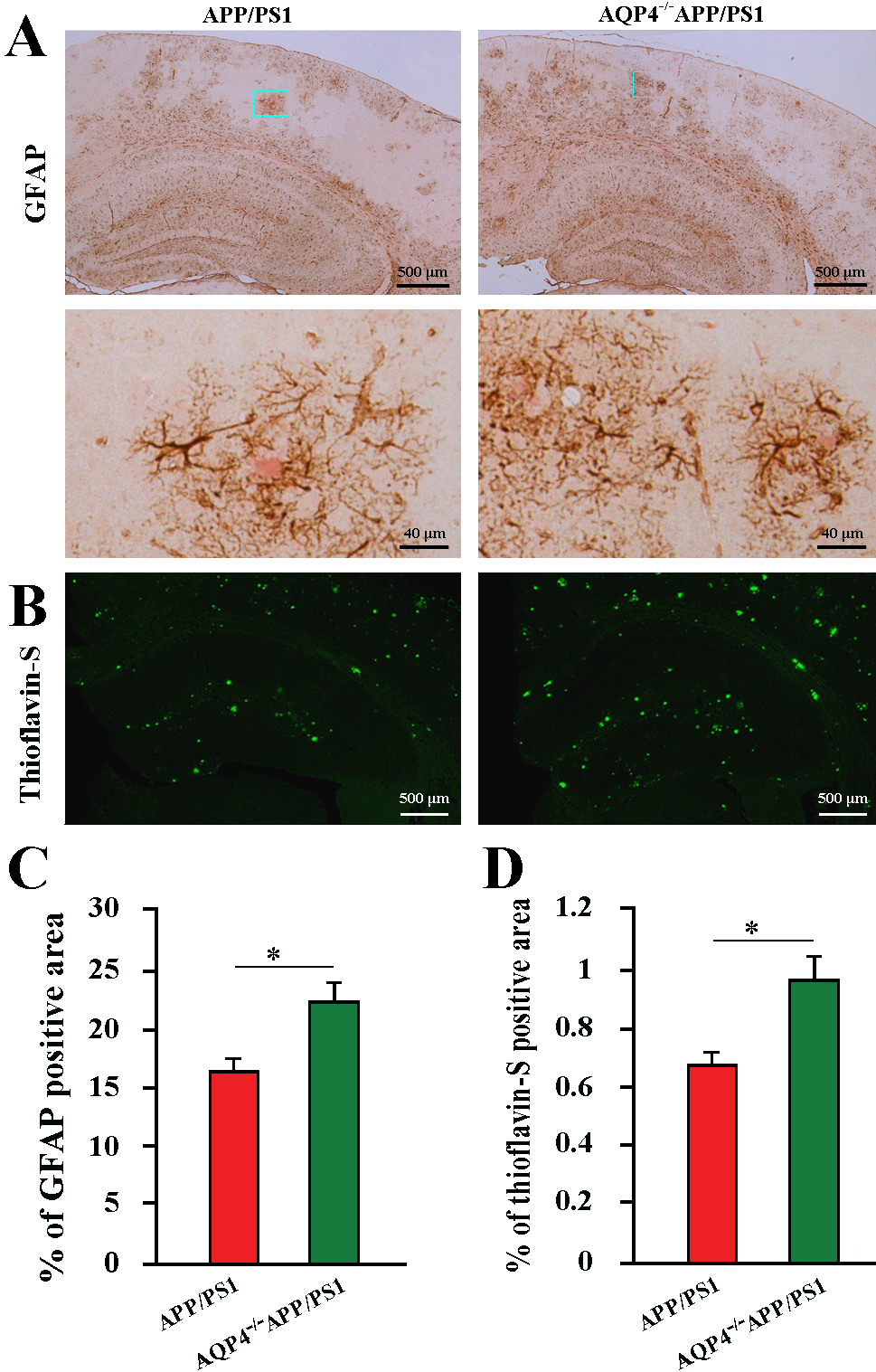

Supplement: Additional file 2: Figure S2. — AQP4 deficiency increased reactive astrogliosis and Aβ plaque deposits in 6.5 month-old APP/PS1 mice. (A) GFAP immunostaining counterstained with Congo Red. AQP4−/−APP/PS1 mice had more extensive reactive astrogliosis in the cerebral cortex and hippocampus than APP/PS1controls. (B) Thioflavin-S staining showing Aβ load in the cortex and hippocampus. (C) The percentage area of GFAP positive. (D) The percentage area of thioflavin-S positive. Data represent mean ± SEM from 5 mice (3 female, and 2 male) per group and analyzed by Student’s t-test. *P < 0.05. (TIF 4322 kb) [file 13024_2015_56_MOESM2_ESM.tif]

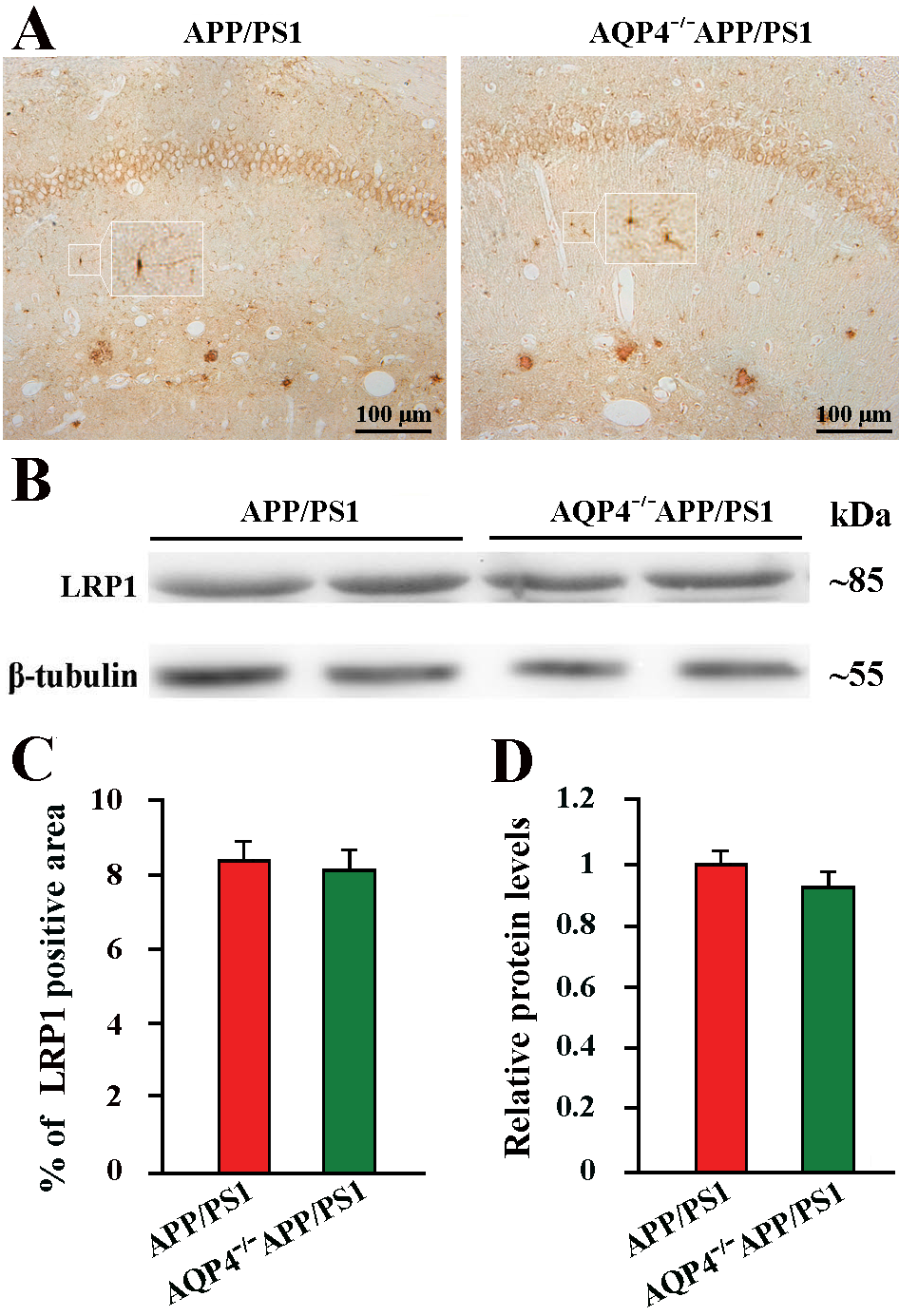

Supplement: Additional file 3: Figure S3. — AQP4 deficiency did not affect LRP1 expression by neurons in APP/PS1 mice. (A) LRP1 immunohistochemistry. High expression of LRP1 was localized to the cell membrane of pyramidal neurons in 12-month old AQP4−/−APP/PS1 mice and APP/PS1 mice. Some glial-like cells (inserted high magnification images) distal to the plaques were also positive for LRP1 in the two genotype mice. (C) Quantitative analysis of the percentage of LRP-1 positive area in the hippocampus and cerebral cortex. (B) Western bolt and (D) densitometry analysis of LRP1 protein levels in the hippocampus and cortex. Data represent mean ± SEM from 5–6 mice (3–4 female, and 1–2 male) per group. The statistical analysis was performed by Student’s t-test. (TIF 3855 kb) [file 13024_2015_56_MOESM3_ESM.tif]

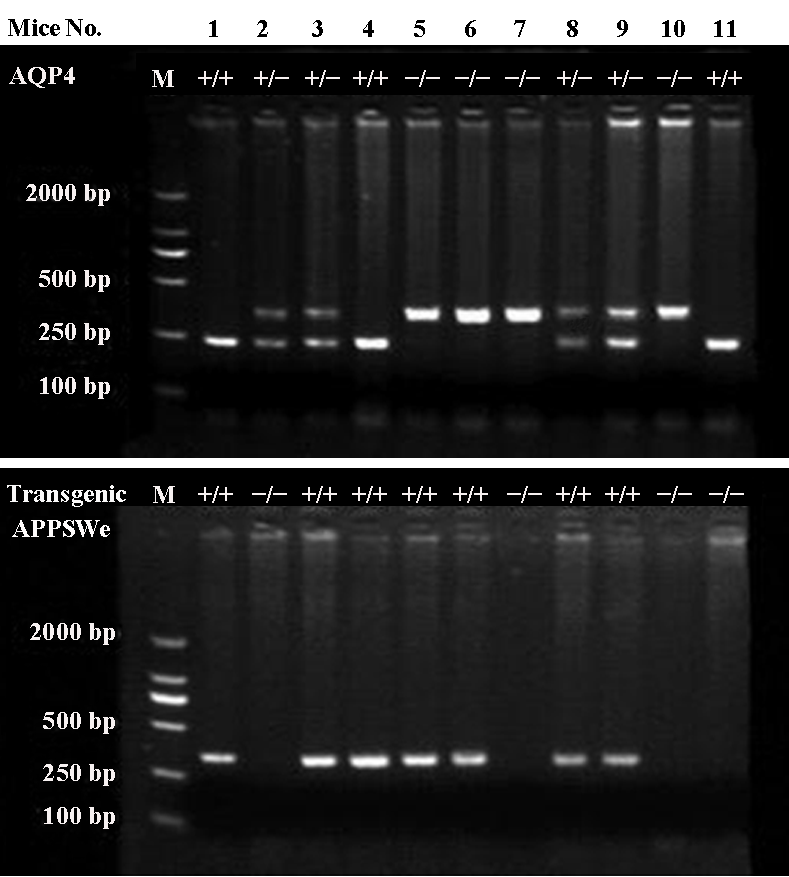

Supplement: Additional file 4: Figure S4. — Genotyping of mice. An example of PCR analysis of AQP4 (the upper panel) and APPSwe (the low panel) mRNA expression in a newborn litter that is generated by AQP4+/−APP/PS1+/+ and AQP4+/−APP/PS1−/− mice. We only detected APPSwe transgene, because APPSwe transgene and PS1ΔE9 transgene are coexpressed under the control of the mouse prion promoter (Jankowsky et al. [60]). APP/PS1 transgene allele yields a 377-bp product; and wild type allele has no product. AQP4 knockout homozygote allele yields a 320-bp product; heterozygote allele yields 240-bp and 320-bp products; and wild type allele yields a 240-bp product. In this litter, eleven mice belong to the following 6 genotypes: AQP4+/+APP/PS1+/+ (mice 1 and 4), AQP4+/−APP/PS1−/− (mouse 2), AQP4+/−APP/PS1+/+ (mice 3, 8 and 9), AQP4−/−APP/PS1+/+ (mice 5 and 6), AQP4−/−APP/PS1−/− (mice 7 and 10), and AQP4+/+APP/PS1−/− (mouse 11). (TIF 2043 kb) [file 13024_2015_56_MOESM4_ESM.tif]
